# International and national frameworks, guidelines, recommendations, and strategies for maternal tobacco prevention and cessation: A scoping review protocol

**DOI:** 10.18332/tid/173088

**Published:** 2023-11-07

**Authors:** Kiran P. Nagdeo, Hyewon Lee, Sarah Forberger

**Affiliations:** 1 Icahn School of Medicine at Mount Sinai, New York City, United States; 2 Dental Research Institute and School of Dentistry, Seoul National University, Seoul, Republic of Korea; 3 Leibniz Institute for Prevention Research and Epidemiology - BIPS, Bremen, Germany

**Keywords:** tobacco control, frameworks, pregnant mothers, maternal health services, smoking cessation

## Abstract

Tobacco use during and around pregnancy can significantly increase the risk of stillbirth, congenital disabilities, premature birth, and low-weight birth. To establish maternal tobacco prevention and cessation frameworks for primary care and dental providers and to facilitate cross-national learning, this scoping review aims: 1) to analyze the body of literature on maternal tobacco prevention and cessation frameworks, guidelines, recommendations, and strategies at the international and national level; 2) to identify common core elements; and 3) to identify gaps in the literature, and propose future initiatives and policy development directions.

A systematic database search based on the JBI methodology and corresponding PRISMA-ScR guidelines will be conducted from January 2015 to August 2023. Searches in different databases will be combined with an expert survey among the members of the World Federation of Public Health Associations (WFPHA) – Oral Health, Tobacco Control, and the Women, Adolescent, and Children’s Working Groups to evaluate the search outcomes and add maternal tobacco prevention and cessation frameworks, guidelines, recommendations, or strategies. Using a systematic review tool to support the screening, two independent reviewers will screen the titles and abstracts of all articles, in order to include the relevant ones for full-text screening, and an independent third author will resolve conflicts, if there is any discrepancy between the two independent reviewers’ search. After a full-text review, data extraction will be conducted for analysis. Descriptive analyses include the publication year, country, legal quality, and target group addressed. A narrative synthesis will describe the scope and content of the frameworks, guidelines, recommendations, and strategies.

The scoping review will serve as a stepping-stone to creating a WFPHA policy resolution on tobacco prevention and cessation framework for women of childbearing age led by the WFPHA Oral Health, Tobacco Control and the Women, Adolescent, and Children’s Working Group members. This WFPHA policy resolution ‘Maternal Tobacco Cessation and Prevention Recommendations for Primary Care Providers and Dental Providers’ will be forwarded to the WFPHA General Council and the General Assembly for approval and will be disseminated to the WFPHA public health association members. Ultimately, this recommendation will be used by each national public health association to consider integrating it into their maternal health strategy.

## INTRODUCTION

### Global tobacco use among women of childbearing age

Around the world, 1.3 billion people use tobacco products, with low- and middle-income nations accounting for 80% of all users^1^. Nicotine, a component of tobacco, is a highly addictive chemical compound, making it difficult to halt the use of any tobacco product containing nicotine^2^. Other potentially hazardous compounds also exist in tobacco or are produced when burned^2^. Tobacco can be smoked, chewed, or snuffed. Cigarettes, cigars, bidis, and kreteks are all forms of smoked tobacco products^2^. Some individuals may use a pipe or hookah (waterpipe) to smoke loose tobacco^2^. Certain organizations, including the US Food and Drug Administration, classify e-cigarettes as tobacco products^3,4^. In response to concerns surrounding vaping-related lung injuries, some individuals may seek novel nicotine delivery methods like heat-not-burn devices, which, although emitting lower levels of harmful chemicals compared to traditional cigarettes, still pose potential cardiovascular health risks due to the presence of substances such as nicotine, particulate matter, benzene, acrolein, and tobacco-specific nitrosamines in their emissions^5^. With the use of smoked tobacco, there is the potential for secondhand smoke exposure, wherein people who do not smoke are exposed to the smoke produced from the burning tip of a cigarette (or other smoked tobacco product) as well as the smoke exhaled by the smoker, as the vapor that smokers exhale typically includes nicotine^3^.

The Lancet Global Health research revealed that smoking while pregnant remains a common practice. Of the 174 countries examined, 29 countries, constituting 17% of the total, had a prevalence of smoking during pregnancy exceeding 10%. Additionally, the study found that over 70% of expectant mothers who smoked during their pregnancies were habitual daily smokers^6^. The Demographic and Health Surveys (DHS) conducted in 54 low- and middle-income countries (LMICs) between 2001 and 2012, which included 58922 pregnant women (aged 15–49 years), found that the prevalence of any tobacco use (smoking and smokeless use) among pregnant women in LMICs was 26% (95% CI: 18–36); South-East Asia had the highest prevalence (51%) and Africa had the lowest at 20% (range: 12–29)^7^. The combined prevalence of current tobacco use among pregnant women varied by location, from 0% (range: 0–3) in Africa to 3% (range: 1–12) in the Western Pacific^7^. Indigenous pregnant women in the US, Canada, Australia, and New Zealand, often consume tobacco at rates two to three times greater than non-indigenous pregnant women^3,8^. Additionally, socioeconomically poor women, women with lower education level, and women with many children are more likely to smoke during pregnancy^3,9^. Along with increased baseline smoking, having a spouse or household member who smokes, being significantly exposed to secondhand smoke, consuming alcohol before or during pregnancy, and experiencing depression, have all been demonstrated to reduce by half the likelihood of smoking cessation while pregnant^3,10^.

### Harmful effects of tobacco use on maternal and child health

Smoking and smokeless tobacco harm expectant mothers and their unborn children^3^. Congenital disabilities, stillbirths, preterm births, and infant fatalities have all been linked to maternal smoking or passive smoking exposure during pregnancy^11^. Due to its detrimental effects on fetal development and pregnant women’s health and well-being, tobacco use during pregnancy is a significant public health issue^12^. Previous research has shown that tobacco use during pregnancy can significantly increase the risks of stillbirth, congenital disorders/disabilities, premature birth, and low-weight birth^11,13^. Children who live with smokers are more likely to acquire respiratory illnesses, including bronchiolitis and pneumonia. They are also more likely to have middle ear illnesses and develop asthma, which can result in hospitalization. They are also more prone to death before turning five. Furthermore, maternal smoking during pregnancy may be a risk factor for retinoblastoma and certain childhood brain tumors. According to a World Health Organization (WHO) report, children exposed to tobacco smoke early in life also have more behavioral issues and perform worse academically^11^. Furthermore, maternal smoking during pregnancy is significantly associated with having a detrimental effect on oral health. WHO’s recent toolkit, for oral health professionals to deliver brief tobacco interventions, discussed that active and passive exposure to tobacco smoke is significantly associated with oral cancer, leukoplakia, periodontal diseases, dental caries, and tooth loss. Smokers are 2 to 4 times at a higher risk of periodontitis, which was also shown to be positively associated with adverse birth outcomes^14-18^. It can also affect the oral health of the baby: maternal smoking during pregnancy can affect tooth development, such as missing teeth in offspring, and is linked to dental caries in children^13,14^.

In a systematic review conducted in 2021, 64 studies were examined to investigate the correlation between maternal smoking during pregnancy and 46 different health conditions^19^. The findings indicated a significantly increased risk association with seven health conditions^19^. Among these, the most substantial risks were observed in cases of spontaneous miscarriage and ectopic pregnancy^19^. No level of fetal tobacco exposure can be considered safe. Smoking during pregnancy can potentially harm the developing fetus, particularly affecting crucial organs like the lungs and brain. Smoking primarily contributes to mortality, disability, and illnesses among newborns^20^. One in five babies born to smoking mothers has a low-weight birth^21^. Moreover, smoking during pregnancy, as well as exposure to secondhand smoke during pregnancy, has been associated with an elevated risk of stillbirth, and both significantly heighten the probability of sudden infant death syndrome (SIDS) (OR=2.98; 95% CI: 2.51– 3.54)^22,23^. Even if no apparent complications affect the fetus or newborn, smoking during pregnancy can still adversely affect the child’s long-term health and development. A systematic review of 79 prospective studies revealed a significant increase in the risk of asthma, ranging 21–85%, with the most pronounced effect observed in children aged <2 years (OR=1.85; 95% CI: 1.35–2.53)^24^. In a prospective cohort study involving 9432 children born in Finland and tracked until aged 15–16 years, 4462 (47.3%) of them became daily smokers during adolescence, finding that maternal smoking during pregnancy had a notable impact on the likelihood of offspring smoking as adolescents^25^.

In several countries, smokeless tobacco is more socially acceptable than smoked tobacco among women and is frequently less expensive than cigarettes^3,7,26^. Smokeless tobacco items include snuff, snus, dip, and regular chewing tobacco. In 21 of the 45 countries with data available, smokeless tobacco was the predominant tobacco product used by pregnant women, according to a recent study focusing on LMICs^3,7^. Using smokeless tobacco while pregnant increases the risk of stillbirth, premature birth, or having an underweight baby. The South-East Asian region had the highest frequency of current smokeless tobacco use among pregnant women (26%), whereas Europe had the lowest prevalence (1%)^7^.

E-cigarettes and other nicotine devices are also dangerous to use during pregnancy, despite their aerosols generally containing fewer hazardous components than cigarette smoke^27^. Moreover, some flavoring used in e-cigarettes may be toxic to an unborn child^27^. Exposure to secondhand smoke during pregnancy is a significant issue.

### Tobacco prevention and cessation efforts for women in childbearing age

The likelihood of ceasing smoking during pregnancy and the prevalence of smoking during pregnancy vary greatly among subpopulations. While many pregnant women are determined to quit smoking and are interested in getting help, they frequently encounter significant barriers, such as a lack of appropriate support from doctors and their healthcare and social environment, but also at an individual level^28-33^.

Pregnancy tobacco prevention and cessation programs have received extensive attention from researchers in high-income nations; however, the work mainly targets individual healthcare providers to support and empower women to change their behavior during pregnancy^34^. Although pregnant women are a highly vulnerable target group, there is a close connection and implicit responsibility for the child through the mother. Therefore, it is necessary to create a comprehensive framework to serve women of childbearing age for tobacco use prevention and cessation. A supportive health provider, social environment, and structural determinants are needed to ensure effectiveness^34^. Therefore, it is crucial to design and execute smoking cessation strategies that prevent these risk factors’ detrimental effects, primarily as they affect children’s health and development^12^.

However, tobacco prevention and cessation measures explicitly tailored to women of reproductive age are not consistently included in maternal health and oral health initiatives and clinical recommendations for primary care and dental practitioners to adapt in their clinical practices. Regarding the contexts, practices, and target healthcare workforces, national maternal tobacco cessation programs vary significantly from country to country. To establish future maternal tobacco control frameworks to facilitate cross-national learning, this scoping review and analysis aims to comprehend the fundamental components of such frameworks.

The objectives of this scoping review are: 1) to analyze the body of literature in a systematic way using specific criteria and databases to search maternal prevention and cessation frameworks, guidelines, recommendations, and strategies at the international and national level; 1) to focus on extracting key information from identified literature to analyze common core elements in such frameworks, guidelines, recommendations, and strategies (e.g. scientific evidence, target workforce, best practices); and 3) to categorize and determine the various resources into overarching themes of tobacco prevention and cessation frameworks, guidelines, recommendations, and strategies for women of childbearing age across the globe to identify gaps in the literature and propose future initiatives and policy development strategies.

## METHODS

The JBI methodology will be adapted for this scoping review^35^ in combination with the PRISMA protocols and checklists. The protocol follows the PRISMA-P checklist for developing and reporting review protocols. The Scoping Review will follow the Preferred Reporting Items for Systematic Reviews and Meta-Analyses (PRISMA): Extension for Scoping Reviews (PRISMA-SCR and flowchart)^25,26^. The checklist covers recommended items to be addressed in the scoping review. We will use a flow diagram for a transparent report of the information flow in the scoping review^25,26^. The flow diagram depicts the flow of information through the different phases of our review. It maps out the number of records identified, included, or excluded, with the reasons for exclusion. We used the PICO (Population, Intervention, Comparator, Outcome) model to inform the research question, the search strategy, and the inclusion and exclusion criteria (Table 1).

**Table 1:** Eligibility criteria based on PICO

*PICO model*	*Concept covered*	*Eligibility criteria*
**Population**	Women of reproductive age (15–49 years) and pregnant mothers with exposure to different types of tobacco [smoking smokeless/chewable tobacco, e-cigarettes, secondhand/thirdhand smoke, snuff, snus, dig, heated tobacco (HNBPs), bidi, etc.]	Articles are eligible that report on: Women aged 15–49 years; Pregnant woman; and/or The general population as it might be that only the article reports a framework that addresses the entire population and does not explicitly address women. In this case, it is checked manually whether women, as defined under a) and b) above, are mentioned as a specific target group in the framework.
**Intervention**	Frameworks, guidelines, recommendations, and strategies at the international and national level addressing tobacco prevention and cessation	Articles that report on frameworks, guidelines, recommendations, or strategies that are population-based interventions with elements of: International-level or national-level approaches; Focus on maternal tobacco prevention and cessation; and Aim to prevent and reduce tobacco consumption in pregnant women or women of reproductive age.
**Comparator**	Not applicable	
**Outcome**	Every outcome reported in the studies regarding the effects of the frameworks, guidelines, and strategies	No eligibility criterion, but any effect that is reported is extracted.

### Eligibility criteria

*Inclusion criteria*

Articles will be eligible for inclusion if they adhere to the criteria shown in Table 1, with all study designs applicable.

*Exclusion criteria*

Research interventions aimed at individual behavioral changes will be excluded as the level of impact must be international or national population-based approaches.

### Languages

The search encompasses English and non-English literature, as journals published in the national language often provide English titles and abstracts to facilitate indexing by literature databases. In the subsequent full-text screening process, studies not published in English will be omitted and documented to track the count of publications excluded due to language limitations.

### Information sources and search

*Databases*

The electronic bibliographic databases that will be searched for this study include MEDLINE (PubMed), Web of Science, Global Health (Ovid), Scopus, CINAHL, and EMBASE for the period January 2015 to August 2023. The Global Strategy for Women’s, Children’s, and Adolescents’ Health (2016–2030) was published in 2015, and serves as a roadmap for ending all preventable maternal, newborn and child deaths, including stillbirths, by 2030 and improving their overall health and well-being^36^. Their evidence-based health interventions for women’s, children’s and adolescents’ health include pre-pregnancy detection and management of risk factors such as tobacco^36^. Therefore, we chose our search period from 2015 and onward. The age range 15–49 years was based on the Demographic Health Surveys^7^. The search strategy for MEDLINE is available in the Supplementary file. The search terms will be adapted for each bibliographic database in combination with the controlled vocabulary and keywords, where these are available.

### Search strategy

*Search string*

An iterative technique will be used to develop the search strategy^28^ adapted from JBI’s three-step approach. A preliminary search has been done in PubMed based on an initial set of key terms. The retrieved articles were reviewed regarding their topical fit. Keywords and synonyms were identified from the retrieved articles and used to revise the search strategy. The final search string will be adapted to other databases. Due to capacity, the grey literature search must be limited to 100 hits. Cohen’s kappa will be calculated to assess the agreement between the two raters. High rates are important to ensuring the validity and generalizability of research findings.

*Grey literature*

Grey literature searches will be conducted on Google Scholar with the following search terms: pregnancy, reproductive age, frameworks, guidelines, recommendations, strategies, tobacco prevention, and tobacco cessation. The first 100 hits are screened regarding the inclusion criteria.

*Additional search*

The results list is complemented by an expert survey among members of the WFPHA Oral Health, Tobacco Control, and Women, Adolescent, and Children’s Workgroup to identify any significant maternal oral health and tobacco control frameworks, guidelines, recommendations, or strategies. This is necessary to identify frameworks, guidelines, and recommendations at the national level that may not have been identified in the systematic and grey literature search. For this purpose, the experts receive a list of the frameworks identified per country with the request to review them and, if necessary, to add country-specific or internationally valid frameworks. The supplemented frameworks are included in the data extraction and added to the flow chart marked accordingly. For the data extraction the experts who have reported the framework are asked to carry out the data extraction. Earlier involvement of the experts in the screening process for non-English literature is impossible due to their limited resources.

*Data management and screening process*

The hits found are exported from the databases and imported into Endnote. EndNote, a program for managing references, and Covidence, an online screening management application, will be used to manage the screening process. In EndNote, duplicate entries will be eliminated, first within each database and then across databases. The de-duplicated dataset is imported into Covidence. Covidence will be utilized for managing the title/abstract and the full-text screening. Titles, abstracts, and full texts will be screened regarding the inclusion and exclusion criteria by two independent screeners. All conflicts are discussed. If no agreement is reached then: 1) the article will be transferred to the full-text screening within the context of the title/abstract screening; and 2) in the case of conflicts in the full-text screening, conflicts are resolved by discussion. If no agreement can be reached, a third person not involved in the screening process will be consulted.

*Data extraction*

Data will be extracted into *a priori* and pre-tested extraction sheets. The extraction sheet used for this purpose will be developed in an iterative process and pre-tested beforehand. A preliminary extraction table covering, for example, details of the article (author, year, country), circumstances, populations, problems, and recommendations is shown in Table 2. The data extraction sheet will be pre-tested on three randomly selected included studies and adjusted if necessary. Two authors will independently extract 10% of the data to ensure quality. If any uncertainties arise, a discussion will be sought within the author group.

**Table 2:** Preliminary data extraction sheet

**Details of reference**	Title	
	Authors	
	Abstract	
	Year of publication	
	Journal	
	Volume	
	Issue	
	Pages	
	DOI	
**Circumstance**	Tobacco uses 1 (direct)	Cigarette Smokeless tobacco E-cigarette Other forms of smoking
	Tobacco uses 2 (indirect)	Secondhand smoke Thirdhand smoke
	WHO Region	African Region, Region of the Americas, South-East Asia Region, European Region, Eastern Mediterranean Region, Western Pacific Region
	Level of frameworks, guidelines, recommendations, strategies	Multi-countries (international) National
	Country/countries	
**Population**	Pregnant Women	Yes or No
	Women of childbearing age (15–49 years)	Yes or No
**Problem**	Problem(s) described?	Yes or No
	Barriers to accessing tobacco counseling mentioned?	Yes or No
	Barriers to implementing tobacco counseling mentioned?	Yes or No
**Recommendation**	Intervention method mentioned?	Yes or No
	Intervention included in the guideline/strategy/recommendation	Examples: Referral Counselling Policy implementation Insurance coverage for tobacco counselling Community engagement Healthcare workforce training Collaboration with non-traditional workforces, such as community health workers, dentists, and others Integration into primary care Integration in non-clinical settings Health communication and health education Digital health Other
	Is the recommended intervention evidence-based?	Yes or No If yes – provide the source of evidence
	If the intervention was implemented, was it evaluated?	Yes or No Examples: Methods to evaluate provider’s competence in providing smoking cessation counseling to pregnant women Methods to evaluate women’s tobacco prevention or cessation outcomes
	Scale of intervention (if implemented)	Local National International
	Other notes	

Since the scoping review aims to analyze the content and scope of the frameworks regarding the target groups and upstream actions, the data extraction consists of three steps and has two purposes: 1) to identify which frameworks are used in the literature and for which evaluation data may be available; and 2) to add to the listed frameworks that have not yet been reported in the literature. Therefore, the data extraction follows three steps:

- All frameworks, guidelines, and recommendations found in the literature are extracted by name. Information is also extracted from the frameworks found in the literature on whether the framework has been evaluated, e.g. study design, sample size, intervention used and effects.
- The list with the names of the frameworks, guidelines, and recommendations is made available to the expert groups, who are asked to complete it based on their knowledge.
- All mentioned frameworks, guidelines and recommendations are searched manually, and the content is extracted from the officially accessible documents. The experts are asked to extract the content for frameworks, guidelines, and recommendations they suggested in languages the author group cannot cover. Data will be collected on the framework’s objectives, legal quality, reach, target audience, and proposed actions to achieve the objectives.

This will provide an overview of the existing frameworks and information on the already evaluated frameworks, with detailed information on evaluation.

### Research synthesis

Results will be synthesized based on the extraction sheet presented related to the review questions and objectives. A narrative synthesis will be used to describe the body of evidence. First, the sources of evidence screened, assessed for eligibility, and included in the review, with reasons for exclusions, will be summarized narratively and with a flow diagram. Further details will be shown, such as publication year, legal quality (law, recommendation, guideline, strategies), level of validity (national/international), and target group to whom the frameworks, guidelines, recommendations, and strategies are addressed, will be conducted. Further, the specifications of tobacco products are addressed in the frameworks, guidelines, recommendations, and strategies reported.

The following items are used to answer the scoping review questions:

1. What is the extent of literature available on maternal tobacco prevention and cessation frameworks, guidelines, recommendations, and strategies at the international and national levels?
2. What are the recurring core elements and characteristics identified within maternal tobacco prevention and cessation frameworks, guidelines, recommendations, and strategies across different sources?
3. What overarching themes can be extracted from the maternal tobacco prevention and cessation literature, and where do gaps in the literature exist?

Various forms of visualization, such as tables and charts, will be used to group the results based on the data we have found. Boundaries for the visualization will depend on the actual extracted evidence.

### Strengths and limitations

Strengths include that the methodology follows the international review standards of Preferred Reporting Items for Systematic Reviews and Meta-Analyses (PRISMA), and an expert survey supplements the systematic literature search. Limitations that we encountered during the preparation of the scoping review will be mentioned (for example, not all frameworks could be found because we do not have an expert from every country in the world and not all frameworks are named in the scientific literature). For example, tobacco prevention and control frameworks, guidelines, recommendations, or strategies, in national language only, which are not covered by experts of the World Federation of Public Health Associations (WFPHA) – Oral Health, Tobacco Control, and the Women, Adolescent, and Children’s Workgroups.

## CONCLUSIONS

The scoping review will serve as a stepping-stone to creating a policy resolution with the expertise of the WFPHA Oral Health, Tobacco Control, and the Women, Adolescent, and Children’s Working Group. The collaboration will create a policy resolution on ‘Maternal Tobacco Cessation and Prevention Recommendations for Primary Care Providers and Dental Providers’, which will be forwarded to the WFPHA General Council and the General Assembly to disseminate to the member Public Health Associations. Ultimately, this recommendation will be considered by each national public health association for integration into their maternal health strategies.

## Data Availability

Data sharing is not applicable to this article as no new data were created.
