# Supplementary material for: International and national frameworks, guidelines, recommendations, and strategies for maternal tobacco prevention and cessation: A scoping review protocol
Source: Tob Induc Dis. 2023 Nov 7;21:144. doi: 10.18332/tid/173088 (PMC10629224; doi:10.18332/tid/173088)
Supplement: Supplementary file 1 [file TID-21-144-s1.pdf]

**Supplement File 1:** Proposed Search Strategy

**MEDLINE**

| | |
|---|---|
| Exposure | "Tobacco Use"/ or Tobacco Smoke Pollution/ or Tobacco Products/ or Tobacco, Smokeless/ **OR** betel nut*.mp. **OR** Tobacco/ or tobacco*.mp. or Tobacco Smoking/ **OR** Smoking/ **OR** e-cigarette.mp. or Electronic Nicotine Delivery Systems/ **OR** Cigarette Smoking/ or cigarette*.mp. **OR** vape*.mp. **OR** vaping*.mp. **OR** vaping/ **OR** kretek*.mp. **OR** bidi*.mp. **OR** Nicotine/ or nicotine*.mp. **OR** second hand smoke*.mp. **OR** "Tobacco Use Disorder"/ or third hand smoke*.mp. **OR** smoking*.mp. **OR** cigar*.mp. **OR** exp smoking devices/ **OR** ecigarette*.mp. **OR** e-cig*.mp. **OR** exp pipe smoking/ **OR** exp tobacco smoking/ **OR** smokers/ **OR** smoker*.mp. **OR** Tobacco Products/ **OR** heet*.mp. **OR** Tobacco/ **OR** heat-not-burn-tobacco*.mp. **OR** hnbt*.mp. **OR** heatstick*.mp. **OR** heated tobacco*.mp. **OR** Chewing tobacco*.mp. **OR** Snuff*.mp. **OR** Snus*.mp. **OR** Dipping tobacco*.mp. **OR** tobacco **OR** "smokeless tobacco" **OR** nicotine **OR** vape **OR** kretek **OR** bidi **OR** "passive smoke" **OR** smoking **OR** smoker **OR** "tobacco products" **OR** heet" "heated tobacco" **OR** snuff **OR** snus **OR** "dipping tobacco" **OR** "chewing tobacco" **OR** "betel nut" |
| Population | Pregnancy/ or pregnan*.mp. **OR** Mothers/ or mothers*.mp. **OR** reproductive age*.mp. **OR** childbearing age*.mp. **OR** prenatal*.mp. **OR** Pregnancy Complications/ or antenatal*.mp. **OR** Pregnant Women/ **OR** birthing person*.mp. **OR** Maternal Health Services/ **OR** Prenatal Care/ or Prenatal Education/ **OR** maternal health*.mp. **OR** reproductive health*.mp. **OR** pre-pregnancy*.mp. **OR** pre-conception*.mp. or Preconception Care/ **OR** Postpartum Period/ or Pregnancy Outcome/ or post-pregnancy*.mp. **OR** postpartum*.mp. **OR** adolescent girl*.mp. or Adolescent/ **OR** teen girl*.mp. |

| | |
|---|---|
| Frameworks | Practice Guideline/ or Guideline/ or guideline*.mp. **OR** framework*.mp. **OR** clinical guideline*.mp. **OR** strategies*.mp. **OR** clinical guidelines*.mp. **OR** consensus*.mp. **OR** recommendation*.mp. **OR** programme*.mp. **OR** program*.mp. OR policy*.mp. **OR** policies*.mp. **OR** recommendat*.ti,kf. **OR** guideline recommendation*.ab. **OR** (guideline* or standards or consensus* or recommendat*).mp. **OR** (position statement* or policy statement* or practice parameter* or best practice*).ti,ab,kf. **OR** (guideline or practice guideline or consensus development conference or consensus development conference, NIH).pt. **OR** guidelines as topic/ |
| Intervention | "Tobacco Use Cessation"/ or Smoking Cessation/ or "Tobacco Use Cessation Devices"/ **OR** Smoking Prevention/ **OR** smoking reduction/ **OR** quit*.mp. **OR** smoking cessation agents/ **OR** nicotine replacement therapy*.mp. **OR** NRT*.mp. **OR** smoking adj2 (cessation or prevention or reduction) |
| Combined with **AND** | |
| | |

© 2023 Nagdeo K.P. et al.
